# Transitioning AI-based sperm assessment from proof-of-concept to clinical practice

**DOI:** 10.1093/hropen/hoag070

**Published:** 2026-08-04

**Authors:** Erica T Y Leung, Xianghan Mei, Brayden K M Lee, Kevin K W Lam, Cheuk-Lun Lee, Raymond H W Li, Ernest H Y Ng, William S B Yeung, Lequan Yu, Philip C N Chiu

**Affiliations:** Department of Obstetrics and Gynaecology, School of Clinical Medicine, Li Ka Shing Faculty of Medicine, The University of Hong Kong, Hong Kong, China; Department of Statistics and Actuarial Science, The University of Hong Kong, Hong Kong, China; Department of Obstetrics and Gynaecology, School of Clinical Medicine, Li Ka Shing Faculty of Medicine, The University of Hong Kong, Hong Kong, China; Department of Obstetrics and Gynaecology, School of Clinical Medicine, Li Ka Shing Faculty of Medicine, The University of Hong Kong, Hong Kong, China; Department of Obstetrics and Gynaecology, Queen Mary Hospital, Hong Kong, China; Department of Health Technology and Informatics, The Hong Kong Polytechnic University, Hong Kong, China; Department of Obstetrics and Gynaecology, School of Clinical Medicine, Li Ka Shing Faculty of Medicine, The University of Hong Kong, Hong Kong, China; Department of Obstetrics and Gynaecology, Queen Mary Hospital, Hong Kong, China; Department of Obstetrics and Gynaecology, School of Clinical Medicine, Li Ka Shing Faculty of Medicine, The University of Hong Kong, Hong Kong, China; Department of Obstetrics and Gynaecology, Queen Mary Hospital, Hong Kong, China; Department of Obstetrics and Gynaecology, School of Clinical Medicine, Li Ka Shing Faculty of Medicine, The University of Hong Kong, Hong Kong, China; Department of Obstetrics and Gynaecology, Queen Mary Hospital, Hong Kong, China; Hong Kong-Shenzhen Key Laboratory of Fertility Regulation, The University of Hong Kong-Shenzhen Hospital, Shenzhen, China; Department of Statistics and Actuarial Science, The University of Hong Kong, Hong Kong, China; Department of Obstetrics and Gynaecology, School of Clinical Medicine, Li Ka Shing Faculty of Medicine, The University of Hong Kong, Hong Kong, China; Hong Kong-Shenzhen Key Laboratory of Fertility Regulation, The University of Hong Kong-Shenzhen Hospital, Shenzhen, China

Dear Editor,

We thank Dr Wang *et al.* for their thoughtful and constructive comments (Wang *et al*., 2026) on our study (Leung *et al*., 2025). We fully agree that transitioning AI-based sperm assessment into clinical practice requires rigorous attention to shortcut learning, clustered validation, and domain shift. Importantly, our study was designed as a proof-of-concept to demonstrate that sperm images contain morphological features linked to zona pellucida (ZP)-binding ability and IVF outcomes. We would like to emphasize that it was not presented as a fully developed, universally deployable clinical tool. We are delighted to take this opportunity to elaborate on how our current methodologies and ongoing work will address these challenges and to outline our framework for prospective external validation.

In our study (Leung *et al*., 2025), variations in sample collection between the ZP-bound and ZP-unbound sperm subpopulations introduced subtle environmental differences in the images. Specifically, the ZP-bound spermatozoa were acquired in a laboratory assay, while the ZP-unbound spermatozoa were sourced from clinical samples. Although these variations are imperceptible to the human eye, they may introduce domain shifts that could affect the model’s generalizability. To mitigate this discrepancy, we used the CycleGAN model to normalize the image quality across both cohorts, minimizing background noise and debris. Saliency map analysis confirmed that this approach significantly enhanced the model’s focus: 91.6% of pixel importance was concentrated on spermatozoa in the CycleGAN-converted images, compared to only 51.4% in the unconverted images, where the remaining 48.6% of attention was diverted to background noise and debris. Although these adjustments mitigated confounding factors, they may not entirely eliminate residual domain-specific biases. To investigate this further, we plan to conduct shortcut-learning experiments using background-only controls, sperm-only images, and CycleGAN ablation studies. These analyses will help determine whether batch-, slide-, or workflow-specific features inadvertently influence the model’s learned representations, thereby helping us enhance its robustness and minimize the encoding of irrelevant features.

Regarding clustered validation, we appreciate Wang *et al*.’s (2026) emphasis on differentiating between patient-level and image-level data splitting. While our initial 5-fold cross-validation used an image-level split on the baseline dataset of 1083 images, we strictly validated the final model on an independent set of 220 images completely withheld from training and optimization. To prevent data leakage and artificially inflated performance, we also evaluated the model’s classification performance at the patient level using independent clinical data. Specifically, when tested on an independent clinical cohort of 117 patients (comprising > 33 000 previously unseen images), the model’s predicted ZP-binding percentages correlated strongly with clinical fertilization rates across low, intermediate, and high fertilization groups (*P* < 0.05) (Leung *et al*., 2025). Although these results suggest a low likelihood of data memorization, we cannot entirely rule out residual center-specific, workflow-related, or batch effects, since all baseline data originated from a single clinical center. Nonetheless, the model demonstrated high reproducibility across multiple individual smears from the same patient sample, indicating that it captures reliable biological features rather than slide- or batch-specific artifacts. In our upcoming prospective trial, we will implement rigorous data-splitting protocols, including leave-one-donor-out, leave-one-slide-out, and leave-one-staining-batch-out cross-validation, to further enhance clinical robustness and real-world generalizability.

To systematically mitigate domain-specific bias, we are integrating a framework based on Cyto-Morphology Adversarial Distillation (CytoMAD) (Lo *et al*., 2024). CytoMAD applies a multi-task adversarial learning strategy to the latent feature space, training the feature extractor to isolate core biological features (i.e. sperm morphology) independent of batch origin. By explicitly decoupling morphological features from batch effects, the model learns to disregard confounding technical and environmental variables, such as variations in staining protocols, image resolutions, and microscope optics. We have trained and validated this architecture using a new, heterogeneous dataset of sperm images captured across diverse microscopy setups and resolutions.

Finally, we acknowledge the single-center limitation highlighted by Wang *et al*. (2026). Because our baseline dataset was derived exclusively from a single clinic (Queen Mary Hospital, Hong Kong), its external generalizability remains to be fully established. To address this and avoid overstating clinical applicability, we are launching a large-scale, prospective, multicenter clinical trial. This expanded dataset will allow us to validate and refine our CytoMAD-integrated model, improving its classification accuracy and robustness across diverse patient populations and laboratory settings. In subgroup analyses, patient data will be stratified by clinical parameters such as cycle number, fertilization rates, and clinical pregnancy outcomes. Furthermore, by implementing standard operating procedures for both sample preparation and the CytoMAD workflow, we aim to standardize our core processes and minimize interlaboratory variability. This approach helps ensure that the model captures genuine biological features rather than technical noise. Consequently, our proposed clinical threshold of 4.9% should be viewed as an internal benchmark specific to our original dataset and imaging setup. We aim to establish a refined, universal clinical threshold using the representative multicenter validation cohort to maximize its utility in real-world clinical practice.

In summary, we appreciate this opportunity to clarify that while our study offers a valuable proof-of-concept for functional sperm image analysis, it is not yet a universally applicable clinical classifier. The critical next step is to demonstrate that AI-predicted ZP-binding remains robust across different patients, slides, staining batches, laboratories, imaging systems, and clinical workflows.
